# Antioxidant potential of biflavonoid attenuates hyperglycemia by modulating the carbohydrate metabolic enzymes in high fat diet/streptozotocin induced diabetic rats

**DOI:** 10.1080/13510002.2020.1722914

**Published:** 2020-02-04

**Authors:** Sundaram Ramalingam, Muthu Karuppiah, Muthusamy Thiruppathi, Shanthi Palanivelu, Sachdanandam Panchanatham

**Affiliations:** a Department of Medical Biochemistry, University of Madras, Chennai, India; b Department of Biochemistry, Saveetha Dental College & Hospital, Saveetha Institute of Medical &Technical Sciences, Saveetha University, Chennai, India; c Department of Chemistry, Manonmaniam Sundaranar University, Tirunelveli, India; d Department of Kinesiology and Nutrition, University of Illinois at Chicago, Chicago, IL, USA; e Department of Pathology, University of Madras, Chennai, India

**Keywords:** *Semecarpus anacardium*, biflavonoid, diabetes, metabolic enzymes, insulin resistant

## Abstract

**Objectives:** The present study was to isolate the biflavonoid (a bimolecular kaemferol structured molecule) and test its efficacy on oxidative stress and carbohydrate metabolic key enzymes in control and high fat diet and streptozotocin -induced diabetic rats.

**Methods:** Type 2 diabetes was induced in male albino wistar rats by feeding them with high fat diet comprising of 84.3% standard laboratory chow, 5% lard, 10% yolk powder, cholesterol 0.2%, and 0.5% bile salt for 2 weeks. After 2 weeks, the animals were kept in an overnight fast and injected with low dose of streptozotocin (35 mg/kg, dissolved in 0.1 M sodium citrate buffer, pH 4.5).

**Results:** At the end of the experimental period, diabetic control rats showed significant increase in plasma glucose, homeostatic model assessment of insulin resistance (HOMA-IR), glycosylated hemoglobin (HbA1c) with concomitant decrease in plasma insulin, total hemoglobin and body weight. The activities of key enzymes of carbohydrate metabolism, lipid peroxidation markers, antioxidant enzymes, glycogen content and glycogen synthase and glycogen phosphorylase were also altered in diabetic rats.

**Discussion:** Oral administration of biflavonoid to diabetic rats significantly ameliorated all the biochemical alterations to near normal levels. The effect produced by the biflavonoid on various parameters was comparable to that of metformin.

## Introduction

Diabetes mellitus has become a major health problem around the world and characterized by metabolic disorders along with several symptoms, including polyuria polydipsia, polyphagia, a selective loss of pancreatic β-cell mass, and a high blood glucose level [1]. Diabetes mellitus can be broadly categorized into type 1 and type 2: Type 2 diabetes mellitus (T2DM) is a frequent form of diabetes representing more than 90% of all diabetic patients [2]. Insulin resistance and impaired insulin secretion are two main characteristics of type 2 diabetes [3]. Insulin resistance in T2DM could be provoked by glucotoxicity, oxidative stress, lipotoxicity and inflammation [4]. Recently, the International Diabetes Federation (IDF) reported that the number of diabetic patients was 415 million in 2015 and is expected to rise to 642 million by 2040 [5]. Chronic hyperglycemia can cause serious complications in different organs. Diet, exercise and several pharmacological agents are treatment approaches for type 2 Diabetes. In view of the side effects associated with the treatment by insulin and synthetic drugs which are available at present, searching for effective and safer hypoglycaemic plant drugs is going on all over the world. Herbal medicines play a vital role in this part to prevent side effects [6].

*Semecarpus anacardium* Linn, belongs to the family Anacardiaceae is distributed in sub-Himalayan region, tropical and central parts of India. *Semecarpus anacardium* nuts are commonly known as marking nut and its vernacular name is ‘Ballataka’ or Bhilwa. It has high priority and applicability in indigenous, Ayurvedic and Siddha system of medicine [7]. Chemical and phytochemical analyses of its nuts revealed the presence of biflavonoids [8] and other phenolic compounds [9], sterols and glycosides [10]. Other components isolated are catechol [11], tetrahydroamentoflavone (THA) [12], jeediflavanone [13], galluflavonone [14], semecarpetin [15] and anacardioflavonone [16] which show various medicinal properties. Some extracts of *S. anacardium* nuts have been found to exhibit antioxidants, anti-inflammatory, antimicrobial and bacterial activities [17, 18]. Recently, catechol derivatives and acyclic isoprenoids have been reported to possess anti-bacterial activity [19, 20]. However, up to date no studies are available on the antidiabetic property of biflavonoid in high fat diet (HFD) and streptozotocin (STZ) – induced type 2 diabetic rats. Therefore, the present investigation was to explore the role of biflavonoid on glucose homeostasis by modulating the carbohydrate metabolic enzymes in HFD/STZ -induced type 2 diabetic rats.

## Materials and methods

### Sources of chemicals

Streptozotocin, high fat diet components such as cholesterol, bile salts, egg yolk power and lard were obtained from Sigma Chemical Company (St. Louis, MO, USA), Sisco Research Laboratories Pvt. Ltd., Mumbai, India, Central Drug House Pvt. Ltd., New Delhi, India, SKM Egg Products Export (India) Limited, Erode, Tamil Nadu, India respectively.Lard was obtained from local market in Chennai. All other chemicals used were of analytical grade.

### Plant material

*Semecarpus anacardium* seeds were purchased from K. R. Vasan Traditional & Herbal Medicine shop, Parris, Chennai, Tamil Nadu, India. The identity of the plant was confirmed by Prof. Raman, plant taxonomist, Centre for Advanced Studies in Botany, University of Madras, Guindy Campus, Chennai – 600025. A voucher specimen (MUCASB- H105) was preserved in the Department herbarium for future reference.

### General experimental procedures

The IR spectra were recorded with a Thermo Satellite FT-IR spectrophotometer. The ^1^H and ^13^C NMR spectra were recorded using 500 and 75.1 MHz Bruker spectrometer with DMSO as solvent and chemical shifts are recorded in parts per million with tetramethylsilane (TMS) as an internal reference. The mass spectrum was obtained from TOFMS mass spectrometers. Column chromatography (CC) was performed on silica gel 60–120 mesh (Merck). Precoated plates of silica gel 60 F_254_ were used for analytical purposes.

## Extraction and isolation

**Figure 1.**
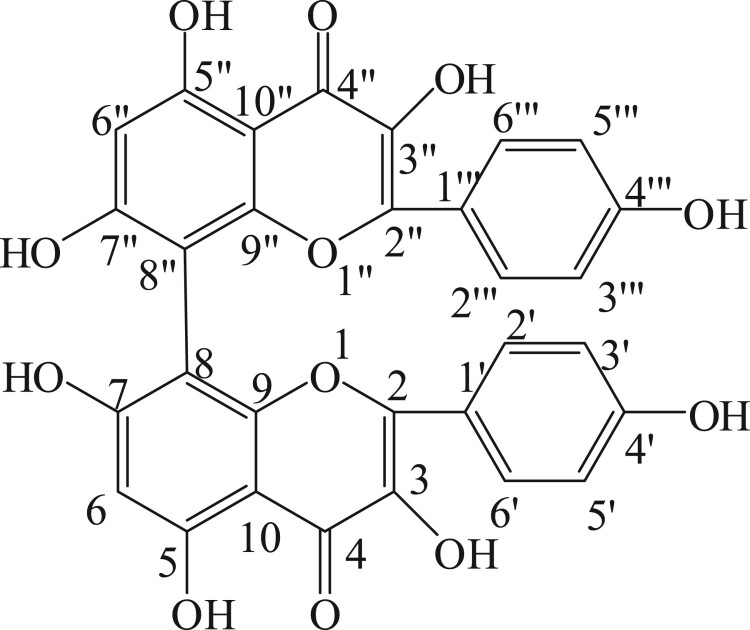
Structure of biflavonoid.

Five hundred grams of *S. anacardium* seeds were bruised and soaked in 2 L of ethyl acetate and kept in refrigerator for 3 days. Then the filtrate was filtered through Whatman filter paper No. 1 and this was repeated three to four times until the filtrate gave no coloration and concentrated using vacuum rotary evaporator at 40°C. The ethyl acetate concentrate was checked on thin layer chromatography with hexane and ethyl acetate in the ratio of 8:2 which showed five spots (compounds). The ethyl acetate extract was chromatographed on silica gel column (Merck 60–120 mesh size) and eluted with hexane and ethyl acetate (80:20 ratio). Fractions (10 ml/ tube) were collected and monitored by thin layer chromatography (pre-coated silica gel Merck-60F254 0.25 mm thick plate). Single spotted fraction (pale yellow color) was collected in clean conical flask and concentrated using vacuum rotary evaporator at 40°C. This process was repeated until getting satisfactory yield of each compound. The structure of the compound was confirmed as biflavonoid on the basis of IR, ^1^HNMR, ^13^C NMR, DEPT and Mass spectral data. The molecular weight of biflavonoid was m/z: 570.46 and molecular formula was C_30_H_18_O_12_. The IR spectra showed the characteristic absorption band of hydroxyl (3423 cm^−1^), the rest of the three spots (three compounds) are under the isolation process. Spectroscopic description of biflavonoid is given in Table 1. The structure of biflavonoid was given in Figure 1. Moreover, we have isolated 5 compounds from *Semecarpus anacardium* seeds. Among these compounds, biflavonoid has shown better antioxidant activity (data not shown) than other compounds because of its structural difference and number of OH group which plays a vital role in scavenging radicals, ie Antioxidant potential because, hydroxyl groups that are prone to donate a hydrogen atom or an electron to a free radical and extended conjugated aromatic system to delocalize an unpaired electron. Therefore, flavonoids, the major factors that determine the radical-scavenging capability [21]. Moreover, antioxidants are the compounds that can delay, inhibit, or prevent the oxidation of oxidizable materials by scavenging free radicals and diminishing oxidative stress. Oxidative stress is an imbalanced state where excessive quantities of reactive oxygen and/or nitrogen species (ROS/RNS, e.g. superoxide anion, hydrogen peroxide, hydroxyl radical, peroxynitrite) overcome endogenous antioxidant capacity, leading to oxidation of a varieties of biomacromolecules, such as enzymes, proteins,DNA and lipids. Oxidative stress is also playing a vital role in the development of chronic degenerative diseases including coronary heart disease, cancer, diabetes and aging [22]. All these informations are encouraged us to chose this drug and evaluate its effect on diabetic rats.

**Table 1.** 1H, ^13^C- NMR and DEPT data for isolated compound biflavonoid (500 MHz, DMSO)

Position	^1^H-NMR	^13^C-NMR	DEPT-135
2(2″)		145.5	
3(3″)		136.2	
4(4″)		176.2	
5(5″)		161.1	
6(6″)	6.40 (s)	98.6	98.6
7(7″)		164.3	
8(2″)		98.6	
9(9″)		148.1	
10(10″)		103.4	
1′(1‴)		116.0	
2′(2‴)	7.68 (*d*, *J* = 5.6Hz)	129.4	129.4
3′(3‴)	6.88 (*d*, *J* = 6.8Hz)	115.5	115.5
4′(4‴)		156.6	
5′(5‴)	6.88 (*d*, *J* = 6.8Hz)	115.5	115.5
6′(6‴)	7.68 (*d*, *J* = 5.6Hz)	129.4	129.4
3-OH(3″-OH)	10.77 (s, br)		
5-OH(5″-OH)	12.49 (s, br)		
7-OH(7″-OH)	9.58 (s, br)		
4′-OH(4‴-OH)	9.36 (s, br)		

## Animals

The male albino rats of Wistar strain with body weight ranging from 180 to 200 g were procured from the Central Animal House Facility University of Madras, Taramani Campus, Chennai, Tamil Nadu, India. They were maintained at an ambient temperature of 25 ± 2°C and 12/12 h of light/dark cycle. Animals were given standard commercial rat chow and water *ad libitum* and housed under standard environmental conditions throughout the study. The laboratory animal protocol used in this study was approved by the Institutional Animal Ethical Committee, University of Madras in accordance with the Indian National law on animal care and use (IAEC No. 01/013/2009).

### Induction of type 2 diabetes in rats

The animals were divided into six groups of six animals each. The rats were fed with high fat diet consisting of 84.3% standard rat chow, 5% lard, 10% yolk powder, 0.2% cholesterol and 0.5% bile salt for 2 weeks [23]. After 2 weeks, the animals were kept in an overnight fast and injected with low dose of streptozotocin (35 mg/kg, dissolved in 0.1 M sodium citrate buffer, pH 4.5) [24]. After three days of STZ injection, overnight fasting blood samples were collected in micro centrifuge tubes containing potassium oxalate and sodium fluoride mixture by pricking the retro orbital sinus with the help of heparinized microhematocrit capillary tubes. Plasma was immediately separated by centrifugation at 3000 × g for 10 minutes and used for estimation of blood glucose. The rats with fasting blood glucose levels above 250 mg/dl were considered diabetic. The HFD/STZ-induced diabetic rats were fed on the high-fat diet for another 4 weeks.

## Experimental design

A total of 42 rats (30 HFD/STZ-induced diabetic rats and 12 normal rats) were used and experimental animals were divided into six groups, each group consists of a minimum of six rats (*n* = 6) detailed as given below. Different concentration of biflavonoid was dissolved in 0.5 ml of olive oil and administered orally using an intragastric tube for a period of 30 days. Metformin was dissolved in 1 ml of double distilled water used as standard drug.

**Table:** 

Group I:	Normal control rats
Group II:	Normal control rats + Biflavonoid (80 mg/kg b.wt)
Group III:	Diabetic control
Group IV:	Diabetic + Biflavonoid (20 mg/kg b.wt)
Group V:	Diabetic + Biflavonoid (40 mg/kg b.wt)
Group VI:	Diabetic + Biflavonoid (80 mg/kg b.wt)
Group VII:	Diabetic + Metformin (500 mg/kg b.wt)

Body weight of all the animals was recorded prior to the treatment and sacrifice. Food and water intake of all groups of animals were monitored on a daily basis for 30 days at a fixed time. Fixed amount of rat chow and fluid was given to each rat and replenished the next day. At the end of the treatment, the rats were fasted overnight, anaesthetized and sacrificed by cervical decapitation. Blood samples were collected in tubes containing potassium oxalate and sodium fluoride (3:1) mixture. Plasma was immediately separated by centrifugation at 3000 × g for 10 minutes and used for estimation of plasma glucose and insulin. The Hb and HbA1c levels were estimated in whole blood samples. The liver tissues were dissected and washed in ice-cold saline and used for carbohydrate metabolic enzymes. All assays were performed with freshly prepared tissue homogenate.

The liver tissues were dissected washed in ice-cold saline and weighed. The liver tissue was minced and homogenized (10%, w/v) with 0.1 M Tris-HCl buffer (pH 7.4) and centrifuged (3000×g for 10 min at 4°C). The resulting supernatant was used for further enzyme assays.

## Biochemical and enzymes assays

### Determination of plasma glucose, insulin, hemoglobin and glycosylated hemoglobin

The level of plasma glucose was estimated spectrophotometrically using commercial diagnostic kit (Agappe Diagnostics Pvt. Ltd., India) Trinder [25]. Hemoglobin was estimated by the cyanmethemoglobin method (Drabkin and Austin [26]. Glycated hemoglobin (HbA1c) was estimated by the method of Sudhakar Nayak and Pattabiraman [27] with modifications according to Bannon [28]. The plasma insulin was measured by the method of Burgi et al. [29].

Determination of Homeostasis Model of Insulin Resistance (HOMA-IR) was measured by the following formula

HOMA−IR=fasting insulin×fasting blood sugar/405

## Preparation of liver homogenates

The freshly prepared liver tissue was minced and homogenized in 0.1 M Tris-HCl buffer (pH 7.4) containing protease inhibitor cocktail (Sigma- Aldrich, Bangalore, India) using a polytron-equipped homogenizer at a fixed low setting on ice. The homogenate was centrifuged at 3000×g for 10 min at 4°C. The resulting supernatant was sampled as a total protein for assay of various enzymes. The protein concentration of the sample was estimated by lowery method using BSA as a standard. The sample was normalized based on protein concentration by including equal amount of total protein.

### Determination of carbohydrate metabolic enzymes

Hepatic hexokinase activity was assayed by the method of Brandstrup et al. [30] The reaction mixture in a total volume of 5.3 ml contained the following, 1 ml of glucose (0.005 M) solution, 0.5 ml of ATP (0.072 M) solution, 0.1 ml of magnesium chloride (0.05 M) solution, 0.4 ml of potassium dihydrogen phosphate (0.0125 M), 0.4 ml of potassium chloride (0.1 M), 0.4 ml of sodium fluoride (0.5 M) and 2.5 ml of Tris–HCl buffer (0.01 M, pH 8.0). The mixture was pre-incubated at 37°C for 5 min. The reaction was initiated by the addition of 1 ml of tissue homogenate. 1 ml of the reaction mixture was immediately transferred to the tubes containing 1 ml of 10% TCA that was considered as zero time. A second aliquot was removed and deproteinised after 30 min incubation at 37°C. The protein precipitate was removed by centrifugation and the residual glucose in the supernatant of tissue homogenate was estimated by the method of Trinder [25] as described previously.

Glucose-6-phosphatase was assayed by the method of Koide and Oda [31]. Incubation mixture contained 0.3 ml of citrate buffer (8.1 M, pH 6.5), 0.5 ml of substrate (0.01 M) and 0.1 ml of tissue homogenate. The reaction mixture was incubated at 37°C for 1 h. Addition of 1 ml of 10% TCA to the reaction tubes terminated the reaction of the enzyme. The suspension was centrifuged and the phosphorus content of the supernatant of tissue homogenate was estimated by the method of Fiske and Subbarow [30,32]. The supernatant was adjusted to known volume. To this 0.5 ml of ammonium molybdate was added followed by 0.2 ml of ANSA. After 15 min the absorbance was read at 640 nm.

Fructose 1,6-bisphosphatase activity was measured by Gancedo and Gancedo [31,33]. The assay mixture in a final volume of 2.3 ml contained 1.5 ml of Tris-HCl buffer (0.1 M, pH 7.0), 0.1 ml of substrate (0.05 M), 0.25 ml of magnesium chloride (0.1 M), 0.1 ml of potassium chloride (0.1 M), 0.25 ml of EDTA (0.001 M) and 0.1 ml of liver homogenate. The incubation was carried out at 37°C for 15 min. The reaction was terminated by adding 1 ml of 10% TCA. The suspension was centrifuged and the supernatant was used for phosphorus estimation by the method of Fiske and Subbarow [32] as described previously.

Glucose 6-phosphate dehydrogenase was assayed by the method of Ellis and Kirkman [32,34]. The incubation mixture contained 0.1 ml of Tris-HCl buffer (0.1 M, pH 8.2), 0.1 ml of magnesium chloride, 0.1 ml of NADP^+^ 0.5 ml of phenazine methosulphate, 0.4 ml of 2,6-dichlorophenol indophenols dye solution and 0.5 ml of liver homogenate. The contents were incubated at 37°C for 10 min. The reaction was initiated by the addition of 0.5 ml of glucose 6-phosphate. The absorbance was read spectrophotometrically at 640 nm against water blank at 1-min intervals for 0 to 5 min.

Glycogen synthase enzyme activity was assayed by the method of Leloir and Goldemberg [33,35]. Briefly, tissues were homogenized in buffer A (pH 8.4) containing 0.25 mmol/L sucrose and 1 mmol/L EDTA using a Polytron-equipped homogenizer at a precise low setting on ice. The homogenate was centrifuged at 25,000 g for 15 minutes at 4°C. The pellet was washed twice in solution containing 0.15 mol/L KCl, 0.01 mol/L Tris buffer, 1 mmol/L EDTA, and 1 mmol/L glucose-6-phosphate and resuspended in buffer A. To determine the activity of glycogen synthase, to 100 μL of each extracted sample, 1.5 μL of 25 mmol/L uridine diphosphoglucose was added and incubated for 10 minutes at 37°C in a 190-μL incubation mixture (4% glycogen, 0.75 mol/L glycine, and 0.05 mol/L glucose-6-phosphate). After incubation, 10 μL of 0.01 mol/L phosphoenolpyruvate and pyruvate kinase (5 U/mL) were added and incubated for 15 minutes at 37°C. At the end of incubation, reaction was arrested by adding 10 μL of 0.1% dinitrophenylhydrazine. The content of the tubes was mixed well and allowed to stand for 5 minutes, and 10 N NaOH was added for the maximum development of color. Afterward, 20 μL ethanol was added and centrifuged for 15 minutes at 750 g. The optical density of the supernatant was measured at 520 nm. The enzyme activity is expressed as micromoles of uridine diphosphate (UDP) formed per minute per milligram protein.

Glycogen phosphorylase enzyme activity was assayed by the method of Cornblath et al. [34,36]. Briefly, tissues were homogenized in buffer (pH 6.8) containing 35 mmol/L glycerol-2-phosphate, 20 mmol/L NaF, and 1 mmol/L EDTA using a Polytron-equipped homogenizer at a precise low setting on ice. The homogenate was centrifuged at 25,000 g for 15 minutes at 4°C. The supernatant was used for enzyme assay. To determine the activity of glycogen phosphorylase, 100 μL of each extracted sample was incubated for 15 minutes at 37°C in a 190-μL incubation mixture (0.05 mol/L glucose-1-phosphate, 2% glycogen, 0.02 mol/L adenosine-5′-monophosphate). At the end of incubation, reaction was arrested by adding 10 μL of 10% trichloroacetic acid (TCA) and centrifuged at 2000 g for 15 minutes at 37°C. To the supernatant, 10 μL of 0.1 mol/L ammonium molybdate and 0.3% 1-amino-2-naphto-4-sulfonic acid were added. After 10 minutes, the optical density of the blue color was measured at 680 nm. The enzyme activity is expressed as micromoles orthophosphate (Pi) liberated per minute per gram protein.

Glycogen was measured by the method of Morales et al. [35,37]. The alkali extract of tissue was prepared by digesting 50 mg of fresh tissue with 3 ml of 30% potassium hydroxide solution in boiling water bath for 15 min. The tubes were cooled and mixed with 5 ml of absolute alcohol and a drop 1 mol/l of ammonium acetate were added to precipitate glycogen and left in the freezer overnight for complete precipitation. Glycogen was collected by centrifugation 2000 g for 20 min. The precipitate was dissolved in distilled water with the help of heating and again the glycogen was re-precipitated by alcohol and 1 mol/l of ammonium acetate and centrifuged. The final precipitate was dissolved in saturated ammonium chloride solution and 4 ml of anthrone reagent was added by cooling the tubes in an ice bath for 20 min. After, cooling the absorbance was read at 640 nm against reagent blank treated in similar manner. Standard glucose (1 mg/ml) solution was also treated similarly.

LPO in liver was estimated by measuring TBARS and hydroperoxides using the methods of Fraga et al. [36,38] and Jiang et al. [37,39], respectively. In brief, 0.1 mL of tissue homogenate was treated with 2 mL of thiobarbituric acid (TBA)–trichloroacetic acid (TCA)–HCl reagent (0.37%TBA, 0.25 M HCl and 15%TCA, 1:1:1 ratio), placed for 15 min in a water bath and then cooled and centrifuged at 3500×g for 10 min at room temperature, the absorbance of clear supernatant was measured at 535 nm against a reference blank. Values were expressed as mM/100 g-tissue. Hydroperoxides were expressed as mM/100 g-tissue. Tissue homogenate (0.1 mL) was treated with 0.9 mL of Fox reagent (88 mg of butylated hydroxy toluene (BHT), 7.6 mg of xylenol orange and 0.8 mg of ammonium iron sulfate were added to 90 mL of methanol and 10 mL of 250 mM sulfuric acid) and incubated at 37°C for 30 min. Then the absorbance was read at 560 nm.

The activity of CAT was estimated by the method of Sinha [38,40]. The reaction mixture (1.5 mL, vol) contained 1.0 mL of 0.01 M phosphate buffer (pH 7.0), 0.1 mL of tissue homogenate and 0.4 mL of 2 M H_2_O_2_. The reaction was stopped by the addition of 2.0 mL of dichromate-acetic acid reagent (5% potassium dichromate and glacial acetic acid were mixed in 1:3 ratio). Then the absorbance was read at 620 nm; CAT activity was expressed as lM of H_2_O_2_ consumed/min/mg protein.

The activity of SOD was assayed by the method of Kakkar et al. [39,41]. 0.5 mL of tissue homogenate was diluted with 1 mL of water. In this mixture, 2.5 mL of ethanol and 1.5 mL of chloroform (all reagents chilled) were added and shaken for 1 min at 4°C then centrifuged. The enzyme activity in the supernatant was determined. The assay mixture contained 1.2 mL of sodium pyrophosphate buffer (0.025 M, pH 8.3), 0.1 mL of 186 lM potassium metabisulfite (PMS), 0.3 mL of 30 lM nitroblue tetrazolium (NBT), 0.2 mL of 780 lM NADH, appropriately diluted enzyme preparation and water in a total volume of 3 mL. Reaction was started by the addition of NADH. After incubation at 30°C for 90 min the reaction was stopped by the addition of 1 mL glacial acetic acid. The reaction mixture was stirred vigorously and shaken with 4 mL of n-butanol. The intensity of the chromogen in the butanol layer was measured at 560 nm against butanol blank. A system devoid of enzyme served as control. One unit of the enzyme activity is defined as the enzyme reaction, which gave 50% inhibition of NBT reduction in one minute under the assay conditions.

The activity of GPX was measured by the method described by Rotruck et al. [40,42]. Briefly, the reaction mixture contained 0.2 mL 0.4 M phosphate buffer (pH 7.0), 0.1 mL 10 mM sodium azide, 0.2 mL tissue homogenized in 0.4 M, phosphate buffer, pH 7.0, 0.2 mL glutathione and 0.1 mL 0.2 mM H_2_O_2_. The contents were incubated for 10 min at 37°C, 0.4 mL 10% TCA was added to stop the reaction and centrifuged at 3200×g for 20 min. The supernatant was assayed for glutathione content using Ellman's reagent (19.8 mg 5,50-dithiobisnitrobenzoic acid (DTNB) in 100 mL 0.1% sodium nitrate). The activity was expressed as µg of GSH consumed/ min/mg protein.

GST activity was determined spectrophotometrically by the method of Habig et al. [41,43]. The reaction mixture contained 1.0 mL 100 mM phosphate buffer (pH 6.5), 0.1 mL 30 mM 1- chloro-2,4-dinitrobenzene (CDNB), and 0.7 mL double distilled water. After pre-incubating the reaction mixture for 5 min at 37°C, the reaction was started by the addition of 0.1 mL tissue homogenate and 0.1 mL of 30 mM glutathione as substrate. After 5 min the absorbance was read at 340 nm. Reaction mixture without the enzyme was used as a blank. The activity of GST is expressed as mM of GSH–CDNB conjugate formed/min/mg protein using an extinction coefficient of 9.6/mM/cm.

Vitamin C was estimated by the method of Omaye et al. [44]. 0.5 mL of tissue homogenate was mixed thoroughly with 1.5 mL of 6% TCA and centrifuged for 10 min at 3500×g. After centrifugation, 0.5 mL of the supernatant was mixed with 0.5 mL of dithiobis- 2-nitrobenzoic acid (DNPH) reagent and allowed to stand at room temperature for an additional 3 h then added 2.5 mL of 85% sulfuric acid and allowed to stand for 30 min. Then the absorbance was read at 530 nm. A set of standards containing 10–50 lg of ascorbic acid were taken and processed similarly along with a blank. Ascorbic acid values are expressed as µM/mg tissue.

Vitamin E was determined by the method of Baker et al. [45]. 0.1 mL of lipid extract, 1.5 mL of ethanol and 2 mL of petroleum ether were added, mixed and centrifuged for 3000 µg for 10 min. The supernatant was evaporated to dryness at 80°C then 0.2 mL of 2,2-1-dipyridyl solution and 0.2 mL of ferric chloride solution was added and mixed well. This was kept in dark for 5 min and added 2 mL of butanol. Then the absorbance was read at 520 nm. Standards of α-tocopherol in the range of 10–100 µg were taken and treated similarly along with blank containing only the reagent. The values are expressed as μM/mg-tissue.

Reduced glutathione was measured according to the method of Beutler and Kelly [46]. The technique involved in protein precipitation by metaphosphoric acid and spectrophotometric assay at 412 nm of the yellow derivative obtained by the reaction of supernatant with 5,50-dithio-bis-2-nitrobenzoic acid (DTNB).

Protein content in the tissue homogenate was determined by the method of Lowry et al. [47]. 0.1 ml of tissue homogenate was made upto 1 ml with distilled water. To this, 4.5 ml of alkaline copper reagent was added, mixed and allowed to stand at room temperature for 10 min. Later, 0.5 ml of Folin's phenol reagent was added and shaken well. The blank and standards were treated in a similar manner. The blue color complex formed was measured at 620 nm after 20 min against the blank.

## Statistical analysis

All the grouped data were statistically evaluated with SPSS\17.0 software. Hypothesis testing methods included one way analysis of variance (ANOVA) followed by least significant difference(LSD) test; P-value of less than 0.05 were considered to indicate statistical significance. All the results were expressed as the mean ± SD for six animals in each group.

## Results

### Dose dependent effect of biflavonoid on blood glucose, insulin and HOMA-IR index

There was a significant increase in the level of plasma glucose and decrease in the level of plasma insulin in HFD/STZ-induced diabetic rats compared to normal control rats. Oral administration of biflavonoid (20, 40 and 80 mg/kg b.wt) daily for a period of 30 days to high fat diet and streptozotocin induced HFD/STZ-induced diabetic rats significantly decreased the level of plasma glucose and significantly increased the level of plasma insulin compared to untreated diabetic rats. In the same context, HFD/STZ-induced diabetic rats showed a significant increase in HOMA-IR when compared with the control rats. Supplementation of biflavonoid significantly decreased HOMA-IR index in a dose dependent manner. The effect was more pronounced at a dose of 80 mg/kg b.wt than 20 and 40 mg/kg b.wt and it was comparable to that of metformin. Therefore, 80 mg/kg body weight was fixed as an effective dose and used for further analysis (Table 2). Control rats treated with biflavonoid (80 mg/kg b.wt) did not show any statistical significant difference on these levels when compared to control rats.

**Table 2.** Dose dependent of biflavonoid on the levels of blood glucose, Insulin and Homeostatic model assessment of insulin resistant of control and experimental animals.

Groups	Glucose (mg/dl)		Insulin ((IU/ml)		HOMA IR	
	Before Treatment	After Treatment	Before Treatment	After Treatment	Before Treatment	After Treatment
Control	85.13 ± 7.17	91.69 ± 6.19	18.66 ± 1.61	19.39 ± 1.72	3.91 ±0.35	4.40 ± 0.58
Normal+ Biflavonoid (80mg/kg b.w)	88.33 ± 7.17	93.22 ±8.80	16.95 ± 1.25	18.00 ± 1.42	3.70 ± 0.43	4.13 ± 0.41
Diabetes Induced	256.83 ± 12.61	306.85 ± 13.10^b^	9.67 ± 11.16	10.12 ±0.89^b^	6.29 ± 0.70	7.67 ± 0.82^b^
Diabetes + Biflavonoid (20mg/kg b.w)	248.17 ±16.03	224.33 ± 12.77^c^	11.16 ± 1.13	12.57 ±0.83^c^	7.09 ± 0.93	6.96 ± 0.53^c^
Diabetes + Biflavonoid (40mg/kg b.w)	232.83 ± 13.01	168.67 ± 14.56^d^	12.13 ± 1.19	14.27 ± 1.45^d^	7.42 ± 0.77	5.97 ± 1.01^d^
Diabetes + Biflavonoid (80mg/kg b.w)	224.83 ± 8.91	130.39 ± 10.12^e^	12.45 ± 1.16	16.86 ± 0.93^e^	7.18 ±1.02	5.42 ± 0.40^e^
Diabetes + Metformin (500mg/kg b.w)	87.33 ±7.30	125.83 ± 6.43f	12.61 ± 0.86	17.30 ±1.17^f^	7.00 ± 0.63	5.38 ± 0.50

Values are given as mean ± SD for six animals in each group.

Values are considered significantly different at *P *< 0.05 with post hoc LSD test *P < 0.05.

The symbols a, b c and d also represent the statistical significance at *p* < 0.05.

^a^ Control vs Drug control rats(normal rats received biflavonoid alone).

^b^ Control vs Diabetic rats.

^c^ Diabetic control rats vs Diabetic rats treated with biflavonoid (20mg/kg).

^d^ Diabetic control rats.vs Diabetic rats treated with biflavonoid (40mg/kg).

^e^ Diabetic control rats.vs Diabetic rats treated with biflavonoid (800mg/kg).

^f^ Diabetic rats treated with biflavonoid (80 mg/kg) vs Diabetic rats treated with Metformin (500mg/kg).

### Effect of biflavoid on body weight gain

**Figure 2.**
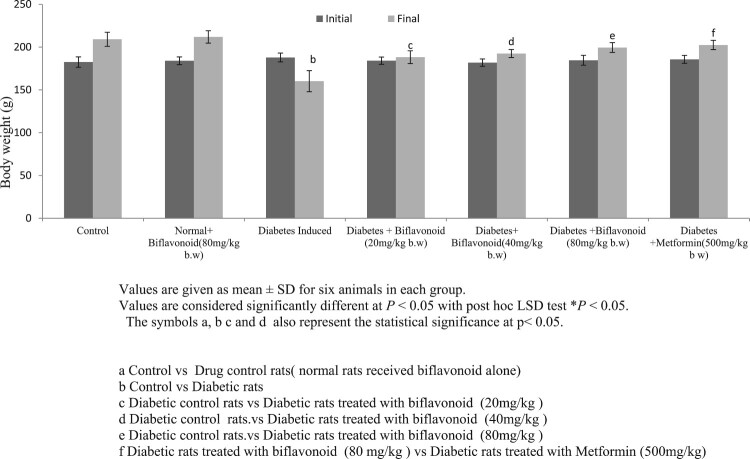
Effect of biflavonoid on body weight gain in control and experimental rats.

Figure 2 depicts the values of the initial and final body weights of the normal and diabetic rats. Body weight significantly decreased in HFD/STZ-induced diabetic rats compared to normal control rats. Oral administration of biflavonoid and metformin to high fat diet and streptozotocin induced HFD/STZ-induced diabetic rats prevents the body weight loss compared to diabetic control rats. No significant changes were observed in biflavonoid alone treated rats.

### Effect of biflavonoid on the levels of hemoglobin and glycated hemoglobin

Table 3 represents the levels of Hb and HbA1c in control and experimental rats. The levels of Hb were significantly decreased whereas the levels of HbA1c were significantly increased in high fat diet and streptozotocin induced HFD/STZ-induced diabetic rats and when treated with biflavonoid and metformin, these values were brought toward near normal level. Whereas the normal rats treated with biflavonoid did not show any significant changes in the levels of Hb and HbA1c.

**Table 3.** Effect of biflavonoid on the levels of hemoglobin and glycated hemoglobin in control and experimental rats.

Group/ Parameters	Hb(g/dl)	HbA1C (%)
Control	12.98±1.12	5.11± 0.45
Normal + Biflavonoid (80mg/kg b w)	13.52±0.84	4.75±0.40
Diabetes Induced	9.20±0.85^b^	12.00±0.86^b^
Diabetes + Biflavonoid (80mg/kg b w)	12.37±0.83^c^	7.13±0.68^c^
Diabetes + Metformin (500mg/kg b w)	12.88±0.73	6.90±0.53

Values are given as mean ± SD for six animals in each group.

Values are considered significantly different at *P* < 0.05 with post hoc LSD test *P < 0.05.

The symbols a, b c and d also represent the statistical significance at *p* < 0.05.

^a^ Control vs Drug control rats(normal rats received biflavonoid alone).

^b^ Control vs Diabetic rats.

^c^ Diabetic control rats.vs Diabetic rats treated with biflavonoid (80mg/kg).

^d^ Diabetic rats treated with biflavonoid (80 mg/kg) vs Diabetic rats treated with Metformin (500mg/kg).

### Effect of biflavonoid on liver and muscle glycogen content

The liver and muscle glycogen content and activities of glycogen synthase and glycogen phosphorylase in normal and diabetic rats (Table 4). There was a significant decrease in liver and muscle glycogen content in high fat diet and streptozotocin induced HFD/STZ-induced diabetic rats when compared to normal control rats. When biflavonoid and metformin were administered to HFD/STZ-induced diabetic rats significantly increased the liver and muscle glycogen content when compared to diabetic control rats.

**Table 4.** Effect of biflavonoid the liver and muscle glycogen content and activities of glycogen synthase and glycogen phosphorylase in normal and diabetic rats.

Group parameters	Liver glycogen (mg/g tissue)	Muscle glycogen (mg/g tissue)	Glycogen synthase (µ mol of UDP formed/h/mg protein)	Glycogen phospharylase (µ mol Pi liberated/h/mg protein)
Control	52.13 ± 5.21	12.00 ± 1.16	760.88 ±35.15	645.43 ±27.09
Normal + Biflavonoid (80mg/kg b w)	53.50 ± 5.02	12.82 ± 1.26	762.84 ± 29.99	647.62 ± 26.84
Diabetes Induced	29.20 ± 2.14^b^	7.14 ± 0.61^b^	424.20 ± 15.25^b^	839.96 ± 31.21^b^
Diabetes + Biflavonoid (80mg/kg b w)	47.34 ± 3.10^c^	10.23 ± 0.53^c^	717.99 ±35.35^c^	636.55 ± 17.02^c^
Diabetes + Metformin (500mg/kg b w)	49.65 ± 4.53	11.01 ± 1.07	719.79 ±36.23	639.04 ± 16.97

Values are given as mean ± SD for six animals in each group.

Values are considered significantly different at *P* < 0.05 with post hoc LSD test *P < 0.05.

The symbols a, b c and d also represent the statistical significance at *p* < 0.05.

^a^ Control vs Drug control rats(normal rats received biflavonoid alone).

^b^ Control vs Diabetic rats.

^c^ Diabetic control rats.vs Diabetic rats treated with biflavonoid (80mg/kg).

^d^ Diabetic rats treated with biflavonoid (80 mg/kg) vs Diabetic rats treated with Metformin (500 mg/kg).

### Effect of biflavonoid on the levels of glycogen synthase and glycogen phosphorylase

The levels of glycogen synthase were significantly decreased whereas the levels of glycogen phosphorylase were significantly increased in the liver of HFD/STZ-induced diabetic rats when compared to normal rats. However, oral administration of biflavonoid and metformin to HFD/STZ-induced diabetic rats reinstated the activities of glycogen synthase and glycogen phosphorylase to near normal levels when compared to untreated diabetic rats (Table 4). There were no significant changes in the levels of these parameters in control rats treated with biflavonoid alone.

### Effect of biflavonoid on glucose metabolic enzymes

Table 5 shows the activities of hepatic hexokinase, glucose-6-phosphate dehydrogenase, glucose-6-phosphatase and fructose-1,6-bisphosphatase of normal and diabetic rats. The activities of hexokinase and glucose- 6-phosphate dehydrogenase were found to be decreased, while the activities of gluconeogenic enzymes: glucose-6-phosphatase and fructose-1,6-bisphosphatase were significantly increased in high fat diet and streptozotocin induced diabetic compared to normal rats. Treatment with biflavonoid and metformin to HFD/STZ-induced diabetic rats reversed the above changes to near normalcy. Normal rats treated with biflavonoid did not show significant changes in the parameters tested.

**Table 5.** Effect of biflavonoid on carbohydrate metabolic enzymes in the liver of control and experimental rats.

Groups	Hexokinase	Glucose 6 phosphatase	Fructose 1,6,bisphasphatase	Glucose 6 phosphate dehydrogenase
Control	217.42 ± 11.39	706.48 ± 16.40	450.91 ±16.40	479.35 ± 10.85
Normal + Biflavonoid (80mg/kg b w)	220.69 ± 9.99	709.70 ± 15.91	453.86 ± 17.12	475.58 ± 11.24
Diabetes Induced	140.38 ± 12.23^b^	1535.19 ± 65.14^b^	750.24 ± 19.44^b^	243.87 ± 22.09^b^
Diabetes + Biflavonoid (80mg/kg b w)	201.71 ±14.34^c^	778.70 ± 52.99^c^	468.50 ± 18.35^c^	450.70 ± 11.63^c^
Diabetes + Metformin (500mg/kg b w)	205.14 ± 15.19	773.94 ± 52.80	464.22 ± 19.23	454.63 ± 15.75

Values are given as mean ± SD for six animals in each group.

Values are considered significantly different at *P* < 0.05 with post hoc LSD test *P < 0.05.

The symbols a, b c and d also represent the statistical significance at *p* < 0.05.

^a^ Control vs Drug control rats(normal rats received biflavonoid alone).

^b^ Control vs Diabetic rats.

^c^ Diabetic control rats.vs Diabetic rats treated with biflavonoid (80mg/kg).

^d^ Diabetic rats treated with biflavonoid (80 mg/kg) vs Diabetic rats treated with Metformin (500 mg/kg).

Units are expressed as: µmoles of glucose-6-phosphate formed/h/mg of protein for hexokinase, µmol of Pi liberated/h/mg of protein for glucose-6- phosphatase and fructose-1, 6-bisphosphatase and µmol of NADPH/minute/mg of protein for glucose-6-phosphate dehydrogenase.

### Measurement of lipid peroxidation markers

Table 6 shows the levels of TBARS and HP in the liver of control and experimental rats. Diabetic rats showed increased levels of TBARS and HP when compared to normal control rats. Oral administration of biflavonoid and metformin to HFD/STZ-induced diabetic rats significantly decreased lipid peroxidation markers in the liver tissues. No significant changes were observed in normal rats treated with biflavonoid alone.

**Table 6.** Effect of biflavonoid on the levels of TBARS and HP in the liver of control and experimental rats.

Groups	TBARS (mmol/g tissue)	Hydroperoxide (mmol/g tissue)
Control	0.81 ±0.07	63.42 ± 5.37
Normal + Biflavonoid (80mg/kg b w)	0.85 ± 0.08	60.79 ± 4.90
Diabetes Induced	1.92 ± 0.11^b^	132.39 ± 9.14^b^
Diabetes + Biflavonoid (80mg/kg b w)	1.25 ± 0.19^c^	75.52 ± 7.39^c^
Diabetes + Metformin (500mg/kg b w)	1.14 ± 0.11	72.10 ± 0.11

Values are given as mean ± SD for six animals in each group.

Values are considered significantly different at *P* < 0.05 with post hoc LSD test *P < 0.05.

The symbols a, b c and d also represent the statistical significance at *p* < 0.05.

^a^ Control vs Drug control rats(normal rats received biflavonoid alone).

^b^ Control vs Diabetic rats.

^c^ Diabetic control rats.vs Diabetic rats treated with biflavonoid (80mg/kg).

^d^ Diabetic rats treated with biflavonoid (80 mg/kg) vs Diabetic rats treated with Metformin (500 mg/kg).

### Measurement of enzymatic and non enzymatic antioxidant enzymes

Tables 7 and 8 illustrate the activities of enzymic antioxidants (SOD, CAT, GPx and GST) and non -enzymic (vitamin C, vitamin E and GSH) antioxidants in the liver and kidney of control and experimental rats. The levels of enzymatic and non enzymatic antioxidant were significantly decreased in diabetic rats when compared to control rats. However, biflavonoid and metformin administration to HFD/STZ-induced diabetic rats significantly improved the activities of the above enzymes. No significant alteration in biflavonoid treated control rats was found with respect to these enzymic and non enzymic antioxidant enzymes levels.

**Table 7.** Effect of biflavonoid on the activities of enzymic antioxidant in the liver of control and experimental rats.

Groups	SOD	Catalase	Glutathione peroxidase	Glutathione-S- transferase
Control	9.04 ± 0.80	81.93 ± 7.62	7.72 ± 0.76	9.17 ± 0.75
Normal + Biflavonoid (80mg/kg b w)	10.35 ± 0.68	84.14 ± 7.58	8.43 ± 0.79	9.84 ± 0.82
Diabetes Induced	3.76 ± 0.34^b^	54.42 ± 3.49^b^	3.49 ± 0.30^b^	4.26 ± 0.38^b^
Diabetes + Biflavonoid (80mg/kg b w)	8.06 ± 0.66^c^	73.12 ± 6.52^c^	6.41 ± 0.52^c^	7.60 ± 0.53^c^
Diabetes + Metformin (500mg/kg b w)	8.76 ± 0.63	75.02 ± 6.40	7.10 ± 0.65	8.33 ± 0.57

Values are considered significantly different at *P* < 0.05 with post hoc LSD test *P < 0.05.

The symbols a, b c and d also represent the statistical significance at *p* < 0.05.

^a^ Control vs Drug control rats(normal rats received biflavonoid alone).

^b^ Control vs Diabetic rats.

^c^ Diabetic control rats.vs Diabetic rats treated with biflavonoid (80mg/kg).

^d^ Diabetic rats treated with biflavonoid (80 mg/kg) vs Diabetic rats treated with Metformin (500 mg/kg).

The activities of enzymes are expressed as follows: SOD – One unit of activity was taken as the enzyme quantity, which gave 50% inhibition of nitroblue tetrazolium reduction in 1 minute/mg protein; CAT – µmoles of H_2_O_2_ consumed/minute; GPx – µg of glutathione consumed/minute/mg protein; GST- µmoles of 1-chloro 2,4-dinitrobenzene-GSH conjugate formed/minute/mg protein.

**Table 8.** Effect of biflavonoid on the activities of non enzymic antioxidant in the liver of control and experimental rats.

Group	Vit C(µmol/mg tissue)	Vit E(µmol/mg tissue)	Reduced glutathione (GSH) (µmol/mg tissue)
Control	2.37 ± 0.23	1.61 ± 0.15	57.34 ± 4.33
Normal + Biflavonoid (80mg/kg b w)	3.02 ± 0.30	2.16 ± 0.20	59.68 ± 4.22
Diabetes Induced Diabetes + Biflavonoid (80mg/kg b w)	1.12 ± 0.12^b^	0.86 ± 0.07^b^	34.16 ± 2.47^b^
	2.17 ± 0.21^c^	1.57 ± 0.11^c^	47.86 ± 3.07^c^
Diabetes + Metformin (500mg/kg b w)	2.58 ± 0.25	1.88 ± 0.15	50.05 ± 3.18

Values are considered significantly different at *P* < 0.05 with post hoc LSD test *P < 0.05.

The symbols a, b c and d also represent the statistical significance at *p* < 0.05.

^a^ Control vs Drug control rats(normal rats received biflavonoid alone).

^b^ Control vs Diabetic rats.

^c^ Diabetic control rats.vs Diabetic rats treated with biflavonoid (80 mg/kg).

^d^ Diabetic rats treated with biflavonoid (80 mg/kg) vs Diabetic rats treated with Metformin (500 mg/kg).

## Discussion

In HFD/STZ-induced diabetic animals, the levels of blood glucose and homeostatic model assessment of insulin resistance (HOMA-IR) were significantly increased whereas the levels of plasma insulin were significantly decreased. However, upon treatment with biflavonoid and metformin, the levels of blood glucose, plasma insulin and HOMA-IR were brought back to near normal. These results clearly indicate that biflavonoid not only bring about glucose lowering action by stimulate the surviving β-cells of islets of Langerhans to release more insulin and also lowered HOMA-IR indices which confirm the restoration of insulin sensitivity through its antioxidant potential because flavonoids are devoted compounds for antioxidant [48,49]. Previous study proved that effects of flavonoids on the survival of beta cells, preservation of pancreatic islet morphology, beta cell mass and beta cell function in STZ induced diabetic mice and STZ/ HFD diabetic rat as well [50]. Moreover, flavonoids decrease beta cell apoptosis and oxidative stress, increase beta cell proliferation/ number and improve glucose tolerance in STZ/HFD diabetic rat and STZ diabetic mice as well [50]. Taken together, it is suggested that biflavonoid may inhibit oxidative stress medicated beta cell apoptosis and stimulates the proliferation of surviving β-cells and its insulin release which leads to restore insulin sensitivity and lower the blood glucose.

Low dose streptozotocin is known to induce rapid destruction of pancreatic β-cells leading to impaired glucose stimulated insulin release and insulin resistance, both of which are marked features of type 2 diabetes [51]. The elevated blood glucose is a result of reduced glucose uptake in muscle and adipose tissue and increased gluconeogenesis, hepatic glucose production and glycogen breakdown [51]. The HFD/STZ-induced diabetic rats revealed signs of polyphagia, polyuria and polydipsia with concomitant reduction of body weight. The decrease in body weight observed in HFD/STZ-induced diabetic rats may be due to an increase in muscle destruction or degradation of structural proteins or due to insulin deficiency the protein content is decreased in muscular tissue by proteolysis [52]. On oral administration of biflavonoid and metformin reinstated the body weight which might be due to an enhancement in glycemic control and increased synthesis of structural proteins.

Glycated hemoglobin (hemoglobin A1c) gives a reliable marker for the estimation of average blood glucose level over 3 months. The marked decrease in total Hb and an increase in HbA1c levels observed in diabetic animals are in accordance with a previous report [52]. In our study, treatment with biflavonoid and metformin decreased the elevation of HbA1c, thereby increasing the level of total Hb in diabetic rats. The decrease in HbA1c levels and increase in total Hb levels might be due to improved glycemic control by biflavonoid.

Glucose is stored in the form of glycogen in the liver and skeletal muscle. The metabolism of glycogen is regulated by glycogen synthase and glycogen phosphorylase [53]. Reduced hepatic glycogen content in HFD/STZ-induced diabetic rats has been attributed to reduced activity of glycogen synthase and increased activity of glycogen phosphorylase. Moreover, glycogen synthesis depends on insulin due to its stimulatory effect. Low insulin and insulin resistance observed in diabetic patients can contribute to decrease in glycogen levels [54]. Our results showed that biflavonoid and metformin treatment produces more insulin from pancreatic β-cells and increase glycogen content in the liver and skeletal muscle of HFD/STZ-induced diabetic rats by increasing the activity of glycogen synthase and inhibiting the activity of glycogen phosphorylase.

Liver plays a central role in blood sugar homeostasis. In diabetic state the activities of hexokinase and glucose 6-phosphate dehydrogenase are decreased which is due to either total absence or insufficiency of insulin [50]. Administration of biflavonoid and metformin to HFD/STZ-induced diabetic rats up regulated the activities of both these enzymes in hepatic tissues via enhanced secretion of insulin which in turn increases the glycogen content in liver and skeletal muscle.

Glucose-6-phosphatase and fructose-1,6-bisphosphatase are the important enzymes in regulating of gluconeogenic pathway. The activities of glucose-6-phosphatase and fructose-1,6-bisphosphatase were increased in the liver of diabetic rats [53]. The increased activities of these two gluconeogenic enzymes from liver may be due to the activation or increased synthesis of the enzymes contributing to the increased glucose production during diabetes by liver. The biflavonoid and metformin treatment may have inhibited the activities of glucose-6-phosphatase and fructose-1, 6-bisphosphatase which in turn enhanced the glucose utilization by increasing the activity of glucose 6 phosphate dyhydrogenase in the liver of diabetic rats. The level of plasma insulin was also found to increase significantly in HFD/STZ-induced diabetic rats treated with biflavonoid which may be a consequence of significant reduction in the level of gluconeogenic enzymes. The reduction in the activities of gluconeogenic enzymes can result in the decreased concentration of glucose in blood. Moreover, previous study has shown that phenolic compound inhibits ATP sensitive K^+^ channels and regulated blood glucose [55]. Phenolic compounds are the family of flavonoids and thereby our compound-biflavonoid is also a flavonoid compound which may have inhibited ATP sensitive K^+^ channels and thereby increasing intracellular calcium ions in the β-cells and stimulating insulin release in the remnant β-cells [56].

In diabetes, tissue damage is considered to be mediated by free radicals by attacking membranes through peroxidation of unsaturated fatty acids [57]. Lipid peroxidation eventually leads to extensive membrane damage and dysfunction [58]. The decreased lipid peroxidation and improved antioxidant status may be one of the mechanisms by which drug treatment could contribute to the prevention of diabetic complications [59]. In the present study the levels of lipid peroxidation and hydroperoxides were significantly increased whereas the levels of enzymic (SOD, CAT, GPx, and GST) and non -enzymic (Vitamin C, vitamin E and GSH) antioxidant enzymes were significantly decreased in the liver of HFD/STZ-induced diabetic rats. However, oral administration of biflavonoid and metformin significantly improved the antioxidant status by reducing the lipid peroxidation in the liver of diabetic rats through its antioxidative and antiperoxidative properties. Previous study proved that kolaviron, a biflavonoid which is structurally similar to our compound – biflavonoid, modulated apoptosis by suppressing oxidative stress and inflammation in diabetes-induced nephrotoxic rats [60]. Furthermore, Ye et al. [61] have already been reported that biflavonoid has anti-inflammatory and analgesic activity in carrageenin-induced paw oedema in rats. This scientific evidence supported our findings.

## Conclusion

In conclusion, the present investigation indicates that biflavonoid treatment restores the levels of plasma glucose, plasma insulin, blood HbA1_C_, blood total Hb, HOMA-IR and the activities of key enzymes involved in the metabolism of glucose and glycogen in HFD/STZ-induced diabetic rats by its antioxidant potential. Further, Glucose transporters (GLUT2&4), Insulin receptor (IR), Insulin receptor substrates (IRS 1&2) and Akt are yet to be tested to confirm the molecular mechanism of actions of biflavonoid in diabetic rats.
